# Correction: Ruan et al. *Roseburia intestinalis* and Its Metabolite Butyrate Inhibit Colitis and Upregulate TLR5 Through the SP3 Signaling Pathway. *Nutrients* 2022, *14*, 3041

**DOI:** 10.3390/nu18213554

**Published:** 2026-10-28

**Authors:** Guangcong Ruan, Minjia Chen, Lu Chen, Fenghua Xu, Zhifeng Xiao, Ailin Yi, Yuting Tian, Yi Ping, Linling Lv, Yi Cheng, Yanling Wei

**Affiliations:** 1 Department of Gastroenterology, Daping Hospital, Army Medical University (Third Military Medical University), Chongqing 400042, China; ruanguangcong@163.com (G.R.); chenminjia525@163.com (M.C.); chenlu130652@163.com (L.C.); xufenghua0603@126.com (F.X.); xiaozf622@126.com (Z.X.); yalyal120@163.com (A.Y.); 15002372386@163.com (Y.T.); pingyi2022@126.com (Y.P.); lvling0827@163.com (L.L.); 2 Department of Pathogenic Biology and Immunology, School of Basic Medicine, Ningxia Medical University, Yinchuan 750004, China

In the original publication [1], there was a mistake in Figure 5E. In the original published version, an inappropriate replicate Western blot image was selected in error for Figure 5E. A more suitable experimental replicate image is adopted herein for replacement. The corrected Figure 5E appears below. The authors state that the scientific conclusions are unaffected. This correction was approved by the Academic Editor. The original publication has also been updated.

## Figures and Tables

**Figure 5:**
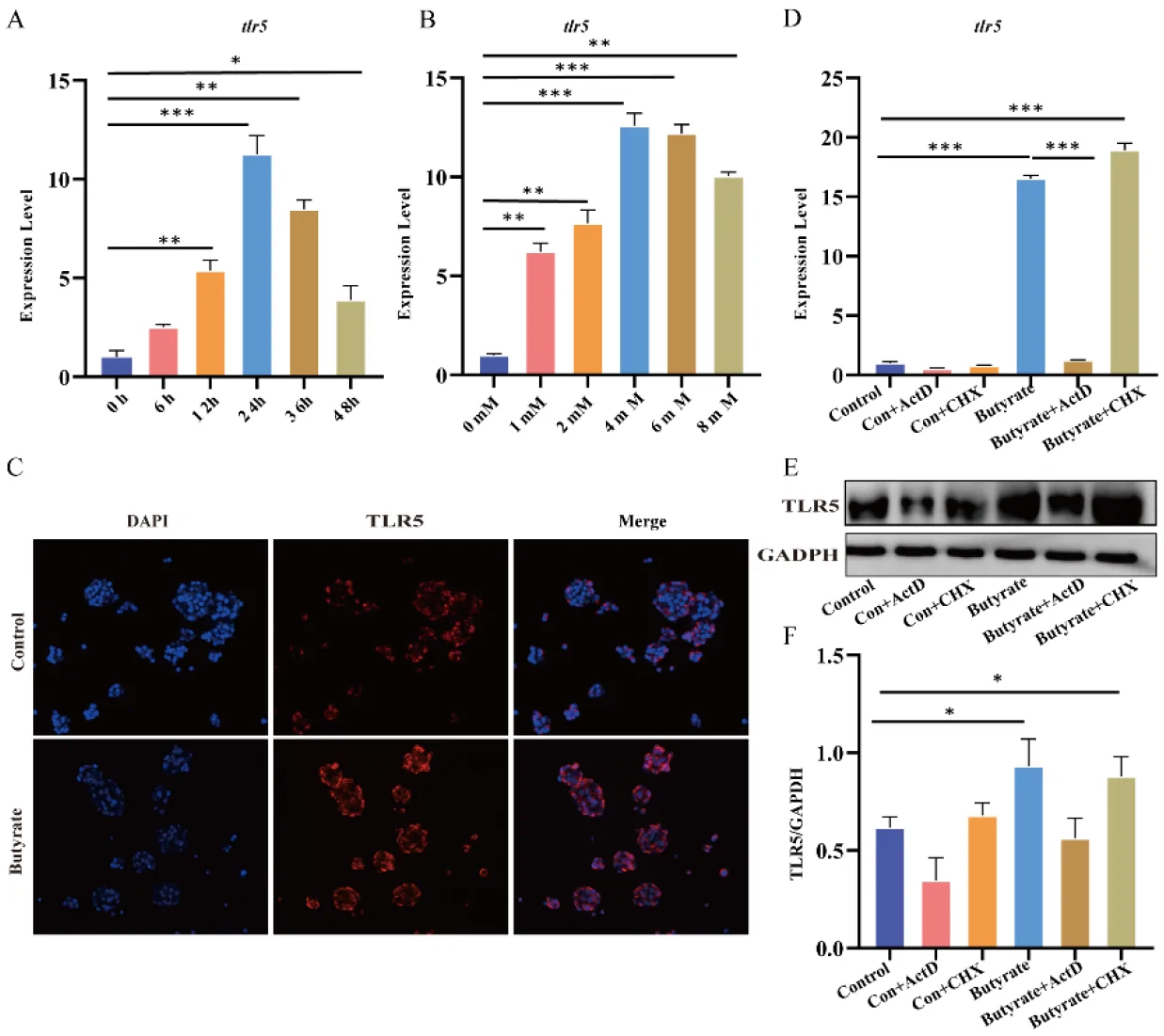
Butyrate increases TLR5 transcriptional expression in HT29 cells. RT–qPCR shows TLR5 mRNA expression in HT29 cells treated with a physiological concentration of butyrate (1 to 8 mM) for 24 h (**A**) or with 4 mM butyrate for a specific duration (**B**). (**C**) Immunofluorescence images of TLR5 in the control and butyrate groups; the butyrate treatment conditions were 4 mM for 24 h. (**D**) The change in TLR5 mRNA expression from adding transcription inhibitors or translation inhibitors in the control and butyrate groups. (**E**) Representative Western blot analysis showing TLR5 expression after the addition of transcription inhibitors or translation inhibitors to the control and butyrate groups. (**F**) Quantitative analysis of Western blot analysis. * *p* < 0.05, ** *p* < 0.01, and *** *p* < 0.001.
